# Robustness and generalizability of a speech‐based digital biomarker derived from recordings of the Clinical Dementia Rating (CDR) interview

**DOI:** 10.1002/alz.094733

**Published:** 2025-01-09

**Authors:** Michael J Spilka, Mengdan Xu, Balazs Toth, Somaye Hashemifar, Rainier Amora, Jessica Robin, Edmond Teng, Cecilia Monteiro, William Simpson

**Affiliations:** ^1^ Winterlight Labs (Cambridge Cognition), Toronto, ON Canada; ^2^ Genentech, Inc., South San Francisco, CA USA

## Abstract

**Background:**

Progressive language changes are established clinical characteristics of Alzheimer’s disease (AD). Advances in Natural Language Processing (NLP) enable more objective, nuanced measurement of language, facilitating the development and validation of speech biomarkers for tracking longitudinal decline in language function. We examined the robustness and generalizability of our previously published speech biomarker score (Robin et al., 2023) in an independent clinical trial dataset.

**Method:**

We analyzed CDR interview recordings at screening, baseline, week 25, and week 49 from 81 English‐speaking mild‐to‐moderate AD patients enrolled in the placebo arm of the Lauriet Phase 2 trial of semorinemab (NCT03828747). We extracted acoustic and linguistic features from the “recent experience” section of the interview, and computed our previously published biomarker score by combining 9 features (6 linguistic, 3 acoustic), derived from recordings from the development dataset (a subset of patients with prodromal‐to‐mild AD from the Tauriel Phase 2 study [NCT03289143]). We then compared the longitudinal trajectory, correlations with clinical endpoints and test‐retest reliability (by computing the difference between screening and baseline recordings) between the development dataset and Lauriet.

**Result:**

Comparison of baseline speech biomarker scores confirmed that the AD population in Lauriet had more severe language dysfunction than the development dataset. The biomarker showed a significant effect of change over time (β = 0.26, p = 0.0003), in line with the development dataset (β = 0.29, p<0.0001). Test‐retest reliability was also comparable (Lauriet ICC = 0.616, p<0.0001; development ICC = 0.50, p<0.0001). Baseline correlations with clinical endpoints were broadly similar (Lauriet vs. development dataset: ADAS‐Cog11 r = 0.22 vs. r = 0.21, CDR‐SB r = 0.24 vs. r = 0.37, ADCS‐ADL r = ‐0.28 vs. r = ‐0.29), while change score correlations showed greater variation (Lauriet vs. development dataset: ADAS‐Cog11 r = 0.37 v. r = 0.17, CDR‐SB r = 0.31 vs. r = 0.45, ADCS‐ADL r = ‐0.35 vs. r = ‐0.22). While the magnitude of individual correlation values differed between datasets, all baseline and change score correlations maintained their direction of association and achieved statistical significance (p’s<0.05).

**Conclusion:**

We replicated prior speech biomarker findings in an independent, more severe AD population, suggesting that the speech characteristics within this score are robust and aligned with clinical progression across disease stages. Additional validation work is ongoing, including the development of comparative biomarkers leveraging the combined dataset.

